# SUGAR Model-Assisted Analysis of Carbon Allocation and Transformation in Tomato Fruit Under Different Water Along With Potassium Conditions

**DOI:** 10.3389/fpls.2020.00712

**Published:** 2020-06-05

**Authors:** Anrong Luo, Shaozhong Kang, Jinliang Chen

**Affiliations:** ^1^Center for Agricultural Water Research in China, China Agricultural University, Beijing, China; ^2^UR 1115 Plantes et Systèmes de Culture Horticoles, INRA, Avignon, France; ^3^UMR 1287 EGFV, Bordeaux Sciences Agro, INRA, Université de Bordeaux, ISVV, Villenave d’Ornon, France

**Keywords:** fruit quality, carbon allocation and transformation, SUGAR model, potassium, water deficit

## Abstract

Carbohydrate concentrations in fruit are closely related to the availability of water and mineral nutrients. Water stress and minerals alter the assimilation, operation, and distribution of carbohydrates, thereby affecting the fruit quality. The SUGAR model was used to investigate the carbon balance in tomato fruit during different growth stages when available water was varied and potassium added. Further, we quantitatively studied the distribution of photoassimilates such as structural carbohydrates, soluble sugars, and starch in fruit and evaluated their response to water and potassium supply. The results revealed that the carbon allocation and transformation dynamically changed during the all growth stages; in fact, variation in carbon content showed similar trends for different water along with potassium treatments, carbon allocation during the early development stages was mainly to starch and structural carbon compounds. The relative rate of carbon conversion of soluble sugars to structural carbon compounds (*k*_3_) and of soluble sugars to starch (*k*_5*m*_) peaked during the initial stage and then dropped during fruit growth and development stages. Carbon was primarily allocated as soluble sugars and starch was converted to soluble sugars at fruit maturation. *k*_3_(*t*) and *k*_5*m*_(*t*) approached zero at the end of the growth stage, mainly due to sugar accumulation. Potassium application can significantly raise carbon flows imported (*C*_*supply*_) from the phloem into the fruit and thus increased carbon allocation to soluble sugars over the entire growth period. Potassium addition during the fruit maturation stage decreased the content of starch and other carbon compounds. Water deficit regulated carbon allocation and increased soluble sugar content but reduced structural carbon content, thereby improving fruit quality.

## Introduction

The quality of agricultural products is an important indicator of evaluating water-saving and efficient agricultural production (Boyd and Barnett, 2011). Carbohydrates formed by photosynthesis play a vital role in fruit production since they are not only the raw materials for fruit growth but also the major determinants of fruit quality (Georgelis et al., 2004; Keller et al., 2008). Furthermore, carbohydrates formed by photosynthesis are closely related to the most basic physiological metabolism of plants, i.e., carbon metabolism (Winter and Huber, 2000). Water and mineral nutrients are important factors affecting fruit carbon metabolism (Buttery et al., 1998). Water stress has been found to be beneficial to the accumulation of hexose, which improves fruit quality (Praxedes et al., 2006). As the element with the largest total absorption into tomatoes, potassium has a wide impact on sugar metabolism in fruits. Fruit soluble sugar content is positively correlated with soil potassium, so increasing the supply of potassium increases sugar accumulation (Zushi and Matsuzoe, 1998); also, potassium is beneficial for increasing the dry matter content and improving fruit quality under drought stress (Manzoor et al., 2018). Potassium can also enhance the transport efficiency of photosynthetic products and thus increase soluble sugar content, thereby upgrading fruit quality (Almeselmani et al., 2009). Interaction between water and potassium promotes sugar accumulation in fruits and so increases fruit sweetness (Feng et al., 2017). Most studies have analyzed the combined effects of water along with potassium on various quality indicators, but there has been little research into how water and potassium together affect carbon allocation and conversion of photoassimilates.

Based on the fruit carbon balance, in 1996, Génard and Souty at French INRA, developed a dynamic simulation of fruit sugar, i.e., the SUGAR model, by considering the physiological mechanisms and the characteristics of fruit sugar metabolism. SUGAR was initially used to describe carbohydrate metabolism by modeling the processes that created and distributed sugar in peach fruit (Génard and Souty, 1996). SUGAR parameters were subsequently modified by Génard to quantitively distinguish the effects of the three physiological processes of assimilate import, sugar metabolism, and water dilution of the sugar content in peach fruit (Génard et al., 2003).

The SUGAR model has been improved since its inception, especially in the areas of estimating sugar content and quantitively modeling carbon allocation in fruit. Dai et al. (2009) used SUGAR to analyze variation in sugar accumulation in response to changes in the source–sink ratio and differences in water supply in grapes. Prudent et al. (2011) used SUGAR with quantitative trait locus analysis (QTL) to identify the basic processes that determined fruit sugar concentration in tomatoes. Wu et al. (2012) also screened a variety of high glucose-to-fructose ratios by the SUGAR model. Dai et al. (2016) identified factors that affect fruit size by investigating the effects of various carbon conversion coefficients.

SUGAR is a widely used model. There have been few studies of the effects of water and mineral nutrients on carbon metabolism. In this study, we analyzed and compared the effects of water and potassium supply on carbon conversion and photoassimilate allocation in tomato fruit. The conclusions of this study provide a theoretical basis for subsequent research into sugar accumulation and improved fruit quality.

## Materials and Methods

### Plant Materials and Growth Conditions

The experiments were conducted in a greenhouse at the Shiyanghe Experimental Station, Gansu Province, Northwest China, from April to August 2017. The greenhouse, 76 × 8 m, was a steel frame construction covered with 0.2 mm thick polyethylene. There was no artificial heating or cooling. A ventilation system on the roof controlled the interior daytime temperature in summer. An indeterminate pink tomato (*Lycopersicon esculentum* Miller cv. Jinpeng 11), which is a commonly planted tomato cultivar in local agriculture, was grown. The temperature of the Shiyanghe experimental station site (37°52′N, 102°50′E, 1581 m elevation) was in the range 14.8–29.1°C from April to August. Precipitation over the period was 164.4 mm, pan evaporation was 2000 mm, and sunshine duration was 3000 h.

At the third to fourth leaf stage, the seedlings were transplanted into plastic containers (top diameter 33 cm, bottom diameter 25 cm, depth 28 cm). Cheesecloth and 1 kg of small gravel were packed at the bottom of each container to prevent soil loss, and the containers were filled with 17 kg of air-dried sandy loam soil (<5 mm) with bulk density 1.3 ± 0.5 g/cm^3^. Volumetric field capacity was 0.25 (cm^3^/cm^3^). Each container was buried up to its top edge in the ground to maintain a soil temperature similar to that in the surrounding field. The soil surface of each container was covered with white polyethylene film to prevent soil water evaporation. The tomato plants were transplanted on 2017-04-26 and harvested on 2017-08-15. The entire growth period, lasting for 111 days, was divided into four growth stages: the vegetative growth stage (2017-04-26–2017-05-13), the flowering and fruit-bearing stage (2017-05-14–2017-06-15), the fruit-swelling stage (2017-06-16–2017-07-13), and the fruit maturation stage (2017-07-14–2017-08-15).

The 240 tomato plants were divided into four groups for the experiment, one control group and three treatment groups, each consisting of 60 plants. The plants in each group were arranged in six north–south rows of ten plants. The plants in each group were given a water treatment and a potassium treatment.

The plants in the control group CK were well-watered in each of the three growth stages. The plants in each of the other three groups T_*i*_, *i* = 1,2,3, were well-watered in two of the three growth stages; in one stage, different for each group, they were subjected to deficit irrigation. Group T_1_ was given half the sufficient water amount in the flowering and fruit-bearing stage (Stage I, 2017-05-14–2017-06-15), group T_2_ was given half the sufficient water amount in the fruit-swelling stage (Stage II, 2017-06-16–2017-07-13), and group T_3_ was given half the sufficient water amount in the fruit maturation stage (Stage III, 2017-07-14–2017-08-15).

In each group, three alternate rows were given a potassium treatment as follows. On 2017-06-01 and 2017-06-05, during the flowering and fruit-bearing Stage I, an amount of potassium was applied to each of the 30 selected pots. The amount of potassium to be applied for optimum fruit development was determined from previous literature to be 0.46 g/kg (K_2_O:soil) per application (Han et al., 2012; Feng et al., 2017). Thus the entire K_2_O application per plant was 15.64 g, and half the plants (30 plants/group, 120 altogether) received this amount of potassium in total. Plants in the control group that were treated with potassium were identified as CKK and plants in the treatment group T_*i*_ that were treated with potassium were identified as T_*i*_K.

The details of the experimental site and layout of the greenhouse are shown in (Supplementary Figure 1).

### Test Items and Methods

#### Irrigation Amount

A 5TE sensor (Decagon Devices, Inc., United States) was installed at 15 cm depth in three randomly selected containers in every treatment to measure soil water content (SWC; cm^3^/cm^3^). The data were collected every 30 min by an EM50 data logger (Decagon Devices, Inc., United States). The sensors were calibrated gravimetrically using sensor-measured data for volumetric water content. When the water content in the containers decreased to 70% of field capacity θ_*f*_ (Agbenin and Tiessen, 1995), which was determined using the cutting ring method (Hu et al., 2011), irrigation was about 95% of field capacity. The amount of irrigation water was calculated using the equation:

(1) $$W=\left(\theta_{t1}-\theta_{t2}\right)\times V$$

where *W* (cm^3^) is the irrigation amount; θ_*t*1_ and θ_*t*2_ (cm^3^/cm^3^) are, respectively, the upper limits of soil water content and the measured soil water content before irrigation; and *V* (cm^3^) is the pot soil volume. To prevent irrigation water leakage from the pots, irrigation should occur over a short period, and the irrigation amount should not exceed field capacity. Irrigation amounts and potassium quantities applied during all the growth stages are given in Table 1.

**TABLE 1:** Details of irrigation amount and potassium application rate for water and potassium treatments during three growth stages.

Treatments	Irrigation amount (mm)				Potassium amount (g K_2_O)	
	Stage I (05/14–06/15)	Stage II (06/16–07/13)	Stage III (07/14–08/15)	Total	Date:06/01	Date:06/05
T_1_	30.33	114.92	91.64	254.97	0	0
T_2_	60.66	57.46	91.64	227.84	0	0
T_3_	60.66	114.92	45.82	239.48	0	0
CK	60.66	114.92	91.64	285.30	0	0
T_1_K	30.33	114.92	91.64	254.97	7.82	7.82
T_2_K	60.66	57.46	91.64	227.84	7.82	7.82
T_3_K	60.66	114.92	45.82	239.48	7.82	7.82
CKK	60.66	114.92	91.64	285.30	7.82	7.82

*Different water treatments (T_1_, T_2_, T_3_, and CK) and different potassium treatments (T_1_K, T_2_K, T_3_K, and CKK) for three stages. Water deficit treatment was divided into three stages: Stage I, from 05/14 to 06/15; Stage II, from 06/16 to 07/13; and Stage III, from 07/14 to 08/15. Equal amounts of potassium were applied during the flowering and fruit-bearing stage (June 1 and 5) for a total of 15.64 g K_2_O.*

#### Index Measurement

Fruits from the first to fourth trusses of the tomato plants were sampled in the experiments, and each treatment was replicated three times. Fruits were picked at 34 days after anthesis (DAA) of the first truss; 37, 48, and 57 DAA of the second truss; 58 and 65 DAA of the third truss; and 66 and 73 DAA of the fourth truss. Since sugar in the fruit is mainly in the form of starch during the early development stage, experimentally measured data from 34 to 73 DAA was used, which included data from stage II (34–57 DAA) and stage III (58–73 DAA). Potassium content was determined by atomic absorption spectrophotometry (Xue et al., 2006). Soluble sugars were extracted using the procedures described in Gomez et al. (2002) and assayed by HPLC analysis. Starch content was determined enzymatically using the method described in Gomez et al. (2003).

#### Statistical Analyses and Drawing

Three-way analysis of variance was performed using R studio version 3.6.1 (Robert, 2016) to evaluate the effects of the three factors irrigation, potassium addition and fruit development stage, and any interactive effects, on the quality index and carbon allocation of tomato fruit. A total of 4 water treatments levels, in which T_1_, T_2_, and T_3_ were water-deficit treatments in comparison to control CK; potassium treatments contained 2 levels: with potassium and without potassium; and two growth stages, stage II and stage III. Moreover, there were two factors during the fruit maturation stage: water and potassium, 4 levels of water and 2 levels of potassium, respectively. Mean values were used (shown by different letters) for water treatments, and the least significant difference (LSD) multiple range test was used to calculate differences between treatments at the confidence level *P* = 0.05. Multiple linear regression, non-linear regression and the Kruskal–Wallis test were all carried out using R, and the ggplot2-based plots were drawn using R package ggpubr (Alboukadel, 2017).

#### Simplified SUGAR Model Description

**FIGURE 1:**
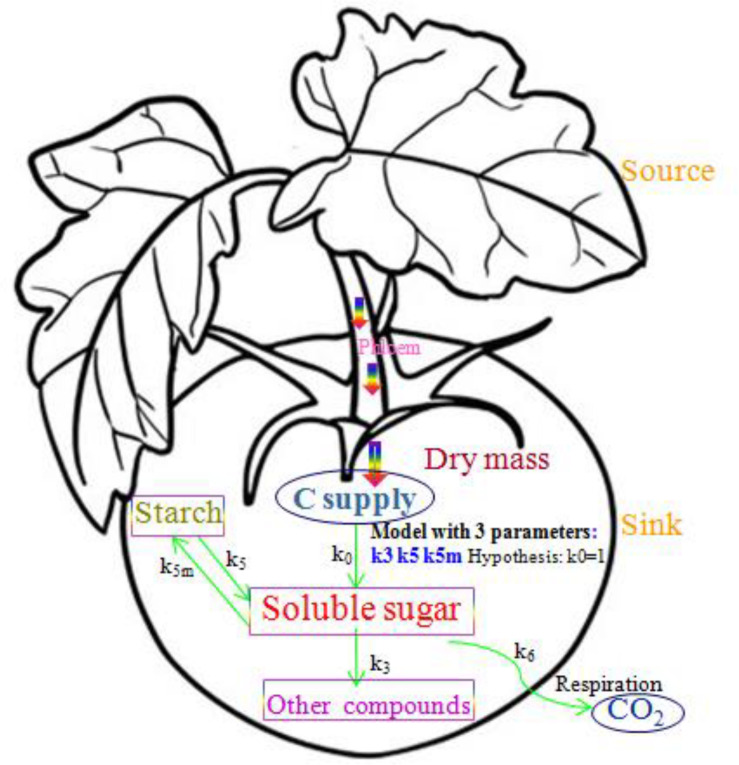
A schematic of the simplified SUGAR model showing sugar metabolism and carbon balance in the tomato fruit. Arrows represent carbon flows. The parameters *k*_3_(*t*), *k*_5_(*t*), and *k*_5*m*_(*t*) are the relative rates of carbon conversion for soluble sugars, starch, and other carbon compounds. Rectangles identify the three major types of carbon compounds in the fruit, and the two ellipses show carbon supply and loss through respiration.

The simplified SUGAR model, which was used to describe the main physiological processes of carbon metabolism in a tomato fruit, is shown in Figure 1. Carbon is mainly supplied to the tomato fruit as sucrose transported by the phloem. Carbon is lost through respiration as CO_2_ derived from the soluble sugars that provide energy and from compounds that form the cellular structure of the fruit (structural carbon) during fruit growth. Remaining carbon is stored in the fruit through carbon metabolism as soluble sugars, starches, and structural carbon compounds such as organic acids, proteins, and cell wall materials. To maintain carbon balance, the conversion of carbon between soluble sugars, starch, and structural carbon compounds must be considered, and compartmentalization of starch in the tomato fruit must be explicitly described (Chen, 2016). In the simplified SUGAR model, only functions *k*_3_(*t*), *k*_5_(*t*), and *k*_5*m*_(*t*) were used to control the conversion of carbon within the fruit, thereby increasing the applicability of the model. The function *k*_6_(*t*) is the fruit respiration rate, which can be directly calculated and is not used as a model parameter. The simplified SUGAR model is represented by the following set of differential equations:

(2) $$\begin{array}{c} \frac{dC_{sol}}{dt}=k_{0}\left(t\right)\frac{dC_{sup}}{dt}+k_{5}\left(t\right)C_{sta}\\ \;-\left(k_{3}\left(t\right)+k_{5m}\left(t\right)+k_{6}\left(t\right)\right)C_{sol} \end{array}$$

(3) $$\frac{dC_{sta}}{dt}=k_{5m}\left(t\right)C_{sol}-k_{5}\left(t\right)C_{sta}$$

(4) $$\frac{dC_{str}}{dt}=k_{3}\left(t\right)C_{sol}$$

(5) $$\frac{dC_{rep}}{dt}=k_{6}\left(t\right)C_{sol}=q_{g}\frac{dDW}{dt}+q_{m}DWQ_{10}^{\left(T-20\right)/10}$$

According to the law of conservation of mass, we obtained:

(6) $$\frac{dC_{DW}}{dt}=\frac{dC_{sol}}{dt}+\frac{dC_{sta}}{dt}+\frac{dC_{str}}{dt}=\frac{dC_{\sup}}{dt}-\frac{dC_{rep}}{dt}$$

Where:

(7) $$\frac{dC_{DW}}{dt}={\text{c}}_{\text{DW}}\frac{dDW}{dt}$$

where *C*_*sol*_, *C*_*sta*_, and *C*_*str*_ (g), respectively, represent the quantities of carbon as soluble sugars, starches, and other structural carbon compounds; *dC*_*sup*_/*dt* and *dC*_*rep*_/*dt* are the carbon flows (g/h) into the fruit (transported by the phloem) and out of the fruit (by respiration); *c*_*DW*_ (g/g C:DW) is the amount of carbon per unit gram of dry mass, which is 0.44 for a tomato fruit (Gary et al., 1998); *DW* (g) is the dry weight of the fruit; *q*_*g*_ is the growth respiration coefficient, which is 0.088 (g/g C:DW) (Gary et al., 1998); *q*_*m*_ is the maintenance respiration coefficient at 20°C and is 0.000 168 (g/g/h C:DW) (Bertin and Heuvelink, 1993); *Q*_10_ is the temperature ratio of maintenance respiration and is 1.4; *T* (°C) is temperature (Bertin and Heuvelink, 1993); *k*_0_ is a dimensionless parameter denoting the assimilates of the fruit that are mainly imported from the phloem as sucrose, assumed to be 1 for a tomato fruit (Bertin and Heuvelink, 1993); *k*_3_(*t*), *k*_5_(*t*), and *k*_5*m*_(*t*), respectively, represent the relative rates of carbon conversion from soluble sugars into other structural carbon compounds, from starch into soluble sugars, and from soluble sugars into starch.

The carbon conversion coefficients *k*_3_(*t*), *k*_5_(*t*), and *k*_5*m*_(*t*) are closely related to the metabolic activity that occurs during the growth and development of the fruit. The rate of starch synthesis is variable, whereas the rate of breakdown is relatively constant (Nguyen-Quoc and Foyer, 2001). This result is supported by the fact that the activity of the enzymes breaking down starch (amylase and starch phosphorylase) varies little during tomato fruit development (Yelle et al., 1988). Therefore, *k*_5_(*t*), which is the rate of conversion of starch to soluble sugars, was considered to be constant (*k*_5_) during fruit growth. Given the dynamic equations for carbon conversion to the three forms (soluble sugars, starch, and structural carbon compounds) equations can be derived from Eqs 3 and 4 to determine *k*_3_(*t*) and *k*_5 *m*_(*t*):

(8) $$k_{3}\left(t\right)=\frac{1}{C_{sol}}\frac{dC_{str}}{dt}$$

(9) $$k_{5m}\left(t\right)=k_{5}\left(t\right)\frac{C_{sta}}{C_{sol}}+\frac{1}{C_{sol}}\frac{dC_{sta}}{dt}$$

The carbon amounts (*C*_*sol*_, *C*_*sta*_, and *C_str_*) and their variation rates (*dC*_*str*_/*dt* and *dC*_*sta*_/*dt*) were calculated by local regression using the data from the experimental measurements. Since *k*_5_(*t*) and *k*_5*m*_(*t*) are functions of each other, the value of *k*_5_ was arbitrarily set when estimating the variation of *k*_5*m*_. Based on the model parameter values obtained from the experimental data, *k*_5_ was 0.296 517 337 and used to calculate *k*_5*m*_(*t*). All parameter values of the SUGAR model were calculated using R functions nls() and optim().

## Results

### Variation of Tomato Fruit Growth and Sugar Concentration

**FIGURE 2:**
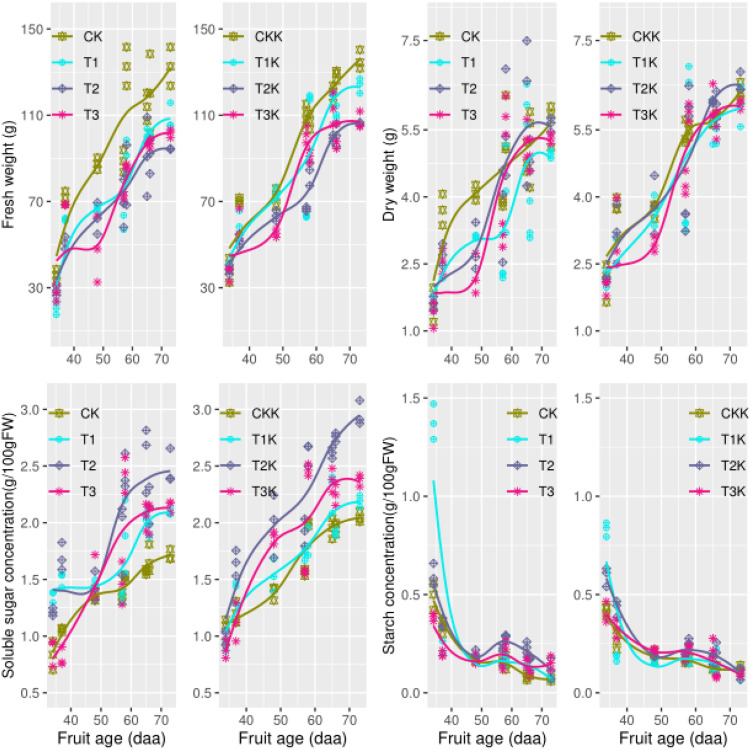
Fruit growth and carbohydrate concentration during all growth stages. Measured fresh weight (points), dry weight(point), and the fitted curves (lines) for different water and potassium treatments are shown as functions of fruit age (days after anthesis, DAA). Variations in the observed data (points) and the fitted curves (lines) for soluble sugar (SSC) and starch (STC) concentrations (g/100 g FW) are shown for different water and potassium treatments. Statistical variables for each treatment are shown in Table 2 for comparison.

Fruit fresh weight (FW) increased gradually as days after anthesis (DAA) increased and reached a maximum at fruit maturation (Figure 2). Mean FW for the water-deficit treatments (T_1_, T_2_, and T_3_) was lower than that for well-watered CK, showing that water had a great effect on FW. The mean FW in potassium treatments (K_1_) was greater than that of the water treatments (K_0_), showing that the potassium had a great effect on FW (Table 2). During the ripening stage, there were considerable differences in FW that corresponded to different water treatments, and similarly, differences in potassium treatments were related to FW. The interactive effects of water along with potassium can be clearly seen in Table 2.

**TABLE 2:** Three-way analysis of variance of FW, DW, SSC, STC, C_sta_, C_sol_, and C_str_ was performed to evaluate the individual and interactive effects of water (4 levels: T_1_, T_2_, T_3_, and CK), potassium (2 levels: K_0_, K_1_) and growth stage (2 levels: stage II and III) on the tomato fruits during the all growth stages and the maturation stage.

Statistics	Treatment	FW (g)	DW (g)	C_sta_ (%)	C_sol_ (%)	C_str_ (%)	SSC (g/100 g FW)	STC (g/100 g FW)
	**All growth stages**							
**LSD method**	**Water treatment (W)**							
	T_1_	76.650b	3.516c	5.370a	29.216a	65.415b	1.701b	0.307a
	T_2_	71.181b	4.132ab	4.923a	30.065a	65.012b	1.987a	0.273a
	T_3_	74.867b	3.834b	4.979a	29.728a	65.294b	1.667b	0.194a
	CK	97.882a	4.313a	4.314a	28.175b	67.511a	1.383c	0.193a
	**Potassium treatment (P)**							
	K_0_	80.145	3.949	4.646	29.296	66.058	1.685	0.242
	K_1_	86.358	4.611	4.253	31.463	64.284	1.855	0.231
	**Growth stage (S)**							
	Stage II	53.611	2.730	6.841	21.772	71.387	1.296	0.370
	Stage III	101.036	5.210	3.015	35.544	61.441	2.054	0.156

**ANOVA**	W	0.0001***	0.017*	0.156	0.046*	0.003**	0.0001***	0.102
	P	0.008**	0.0001***	0.250	0.001***	0.0003***	0.0001***	0.652
	S	0.0001***	0.0001***	0.0001***	0.0001***	0.0001***	0.0001***	0.0001***
	W × P	0.338	0.695	0.813	0.999	0.924	0.227	0.556
	W × S	0.049*	0.210	0.110	0.756	0.093	0.001***	0.011*
	P × S	0.187	0.269	0.372	0.267	0.003**	0.100	0.366
	W × P × S	0.947	0.529	0.580	0.625	0.157	0.583	0.148
	Residuals	263	0.91	5.6	19	11	0.089	0.028

	**Maturation stage**							
**LSD method**	**Water treatment (W)**							
	T_1_	100.159b	4.650b	3.001ab	35.431ab	61.568b	1.987c	0.130bc
	T_2_	89.668c	5.546a	3.336a	36.742a	59.922c	2.412a	0.204a
	T_3_	95.500bc	5.339ab	2.934ab	35.738ab	61.329b	2.171b	0.165ab
	CK	124.814a	5.315ab	2.336b	33.407b	64.257a	1.618d	0.098c
	**Potassium treatment (P)**							
	K_0_	102.535	5.212	2.901	35.330	61.769	2.047	0.149
	K_1_	110.28	5.966	2.757	38.280	58.964	2.317	0.154

**ANOVA**	W	0.0001***	0.027*	0.048*	0.043*	0.002**	0.0001***	0.0001***
	P	0.0001***	0.0001***	0.395	0.0001***	0.0001***	0.0001***	0.649
	W × P	0.006**	0.181	0.185	0.416	0.046*	0.123	0.344
	Residuals	93	0.443	0.687	8.39	5.95	0.029	0.003

Different letters following the mean values of water treatments indicate significant differences at the P < 0.05 level using the LSD method. FW, fruit fresh weight; DW, dry weight; SSC, soluble sugar concentration; STC, starch concentration; C_sta_, carbon in the form of starch; C_sol_, carbon in the form of soluble sugar; C_str_, carbon in the form of the other carbon compounds. The values shown are the average values for the four water treatments T_1_, T_2_, T_3_, and CK, in which T_1_, T_2_, and T_3_ were deficit irrigation during one growth stage only and CK was full irrigation; similarly for potassium treatments: with potassium (K_1_) and without potassium (K_0_); stages, Stage II (34–57 DAA) and Stage III (58–73 DAA). Different letters (a, b, c) following the mean values in water treatments indicate significant differences at the P < 0.05 level. Differences due to the treatment were determined by analysis of variance for S, growth stage; W, water effect; P, potassium effect; W × P, interactive effect of water and potassium; W × S: interactive effect of water and stage; P × S: interactive effect of potassium and stage; W × P × S: interactive effect of water, potassium and stage. *** denotes significant difference at a confidence level of P < 0.001; ** denotes significant difference at a confidence level of P < 0.01; * denotes significant difference at a confidence level of P < 0.05; ns denotes no significant difference.

Fruit dry weight (DW) also increased as DAA increased; it increased rapidly during the early development stage and leveled off during fruit development (Figure 2). Mean DW was ordered by treatment CK > T_2_ > T_3_ > T_1_, and water had a significant effect on DW (Table 2). Potassium treatments (T_1_K, T_2_K, T_3_K, and CKK) resulted in significant increases in DW, as shown by K_1_ > K_0_ in Table 2. All treatments significantly changed DW in different growth stages, and the relationship between growth stage and DW was pronounced. Both water and potassium had considerable influence on DW during the maturation stage (Table 2).

The concentration of soluble sugars (SSC) greatly increased as DAA increased and peaked at fruit maturation (Figure 2). Mean SSC was greater for the water-deficit treatments than for CK, in which T_2_ was the maximum and the effect of water on SSC was pronounced (Table 2). Potassium application resulted in very significant change in SSC, and SSC was greater for K_1_ than for K_0_ (Figure 2 and Table 2). The development stage effect on SSC was really notable, and the interactive effect between water and growth stage was obvious. SSC at the fruit maturation stage differed between water treatments, and potassium had an evident influence.

Starch concentration (STC) increased during the early development stage and then decreased to a very low level (near zero) at maturity (Figure 2). No difference in mean STC was observed between water treatments. However, the relationship between growth stage and STC was extremely remarkable, and the interactive effect between growth stage and water markedly differed. Water had a seriously effect on STC at maturity (Table 2) than during all growth stages.

### Carbon Allocation and Variation in Tomato Fruit

**FIGURE 3:**
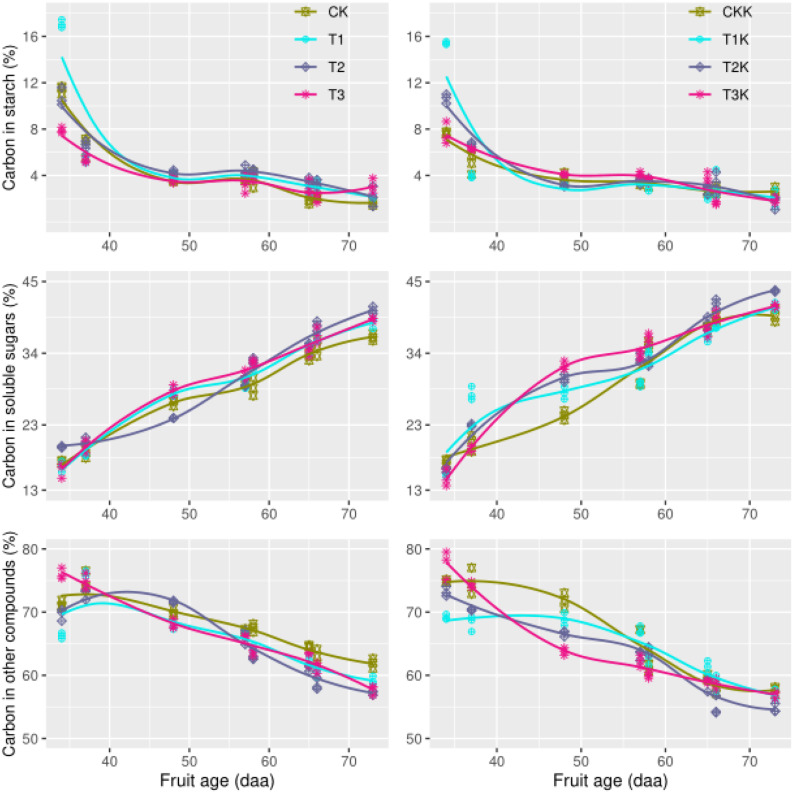
Carbon allocation during all growth stages. Measured values (points) and fitted curves (lines) are shown for different treatments. The graphs show variation in carbon concentrations in the form of starch, soluble sugar, and other carbon compounds.

Carbon content in the form of starch (*C*_*sta*_) varied significantly as DAA increased. The amount of carbon allocated to starch in the sink (*C*_*sta*_) decreased from 16% in the early development stage to 2% at the fruit maturation stage (Figure 3). As the fruit ripening, the declining trend between different water treatments was consistent, and no marked difference was found between water treatments. The variation trend of potassium treatments were similar, and carbon allocation to starch decreased as DAA increased (Figure 3). Neither water nor potassium significantly affected *C*_*sta*_. However, the development stage effect on *C*_*sta*_ was seriously remarkable (Table 2).

Soluble sugar content (*C*_*sol*_) increased as DAA increased (Figure 3). *C*_*sol*_ increased gradually from 13% at the beginning of the fruit growth stage to 45% at harvest, when the fruit was mature. The trend of this variation was consistent across different water treatments, but water-deficit treatments showed greater increases than CK. *C*_*sol*_ for K_1_ was greater than K_0_. The effects of potassium and stage on *C*_*sol*_ were statistically significant (Table 2).

Most carbon is allocated as structural carbohydrates *C*_*str*_. The carbon content of *C*_*str*_ decreased greatly as DAA increased from 75% during the early development stage to about 50% at fruit maturation. This downward trend was consistent between the water-deficit treatments and CK; the mean of *C*_*str*_ for CK was greater than for the water-deficit treatments. Water clearly had a significant effect on *C*_*str*_ (Figure 3 and Table 2). *C_str_* for K_1_ was less than for K_0_. There were large differences in *C*_*str*_ between different development stages, and the interactive effect between potassium and stage was impressive (Table 2).

### Variation of Carbon Conversion Coefficient in Tomato Fruit

Change in *k*_3_(*t*) and *k*_5*m*_(*t*) over the period of the experiment was calculated from the carbon amounts (*C*_*sol*_, *C*_*sta*_, and *C*_*str*_) and their variation rates (*dC_*str*_/dt* and *dC_*sta*_/dt)* using Eqs 8 and 9 with the experimental data. The flux of *C*_*supply*_ was calculated using Eq. 6.

**FIGURE 4:**
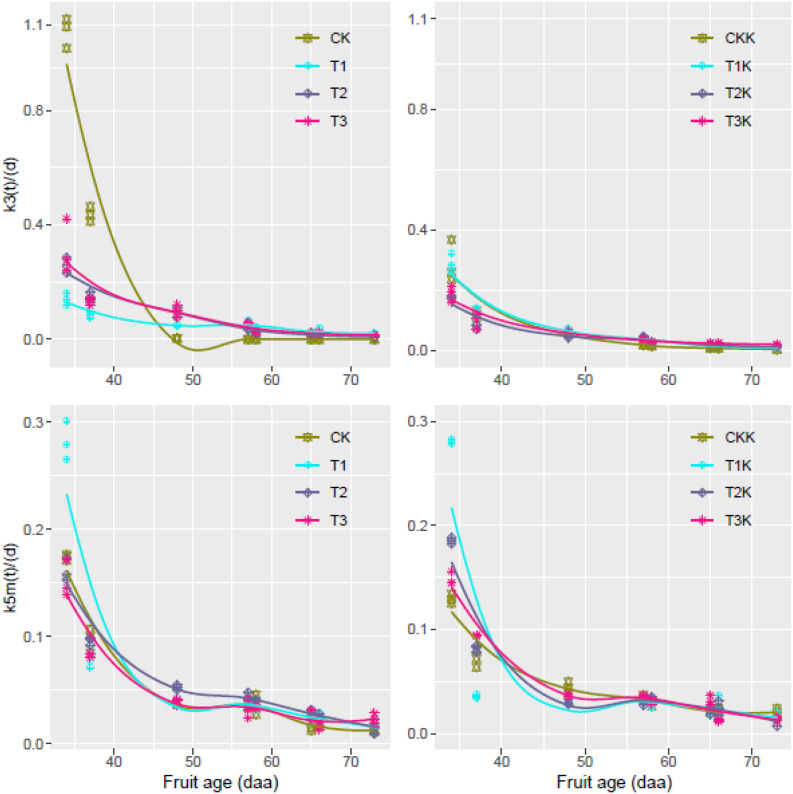
Relative rates of carbon conversion from soluble sugars to other carbon compounds [*k*_3_(*t*)] and from soluble sugars to starch [*k*_5*m*_(*t*)] during the entire growth period; values were calculated from Eqs 8 and 9 using R with the dataset for all treatments; fitted curves (lines) are also shown.

The calculated carbon conversion coefficients *k*_3_(*t*) and *k*_5*m*_(*t*) were plotted against DAA (Figure 4). *k*_3_(*t*) decreased as DAA increased in all treatments; it reached a maximum during the early development stage and then decreased to a very low level (near zero) at maturity. Values of *k*_3_(*t*) for the water-deficit treatments were noticeably less than for CK during the early development stage, and the greatest decrease in *k*_3_(*t*) over the entire growth stage was for CK (Figure 4). The potassium treatments displayed the same trends as the water treatments. *k*_5*m*_(*t*) decreased as DAA increased for all the treatments; it reached a maximum during the early development stage and then decreased to an extremely low level (near zero) at fruit maturation. *k*_5*m*_(*t*) behaved similarly for the potassium treatments. *C*_*supply*_ flux was considerably greater for K_1_ than for K_0_ during the entire growth period (Supplementary Figure 2).

### Carbon Allocation During the Maturation Stage

**FIGURE 5:**
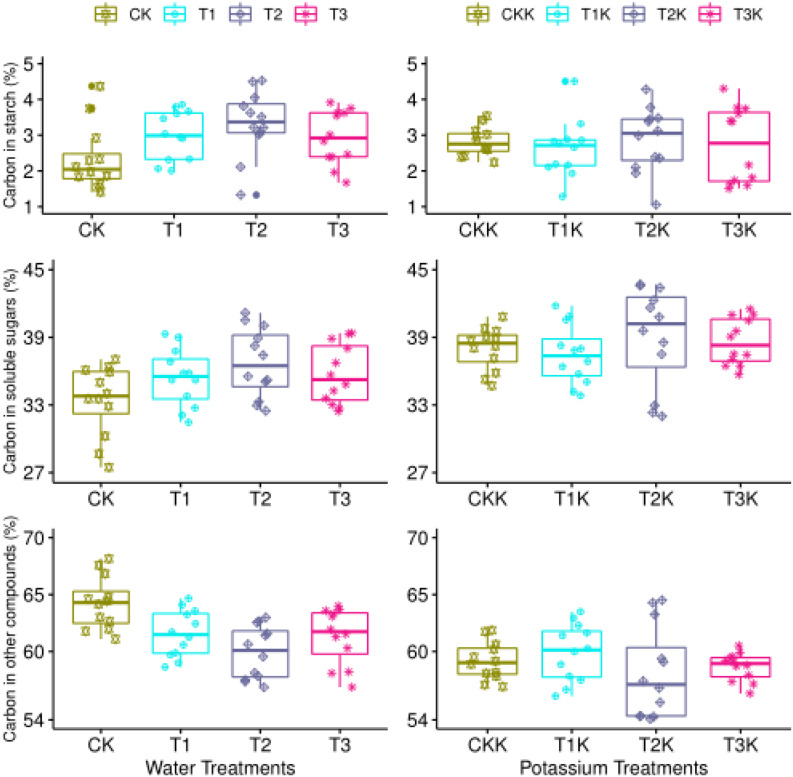
Carbon allocation during the maturation stage. Measured values (points) are shown for different water and potassium treatments. Statistical variables calculated for each treatment are presented in Table 2 for comparison.

The mean value of *C*_*sta*_ for the water treatments was greatest for T_2_ and least for CK, showed that water had a considerable influence (Table 2). The *C*_*sta*_ for K_1_was lower than for K_0_, but the effect of potassium showed no notable change (Figure 5 and Table 2).

The mean value of *C*_*sol*_ was significantly greater for the water-deficit treatments (T_1_, T_2_, and T_3_) than for CK during the mature stage. T_2_ had the maximum mean value of *C*_*sol*_ (Table 2). *C*_*sol*_ was clearly greater for K_1_ than for K_0_ (Figure 5 and Table 2).

The mean value of *C*_*str*_ for the water-deficit treatments was noticeably less than for CK (Figure 5). There were significant differences between water treatments, but not between T_1_ and T_3_ (Table 2). The *C_str_* for K_1_ was lower than for K_0_ (Figure 5 and Table 2). Water had an influence on *C*_*sta*_, but potassium did not. Water and potassium significantly affected *C*_*sol*_. Water also had a noteworthy impact on *C*_*str*_, and potassium significantly affected *C*_*str*_ at maturaty. The interactive effect of water and potassium on *C*_*str*_ was also significant (Table 2).

### Changes in *k*_3_(*t*) and *k*_5*m*_(*t*) at Fruit Maturation

**FIGURE 6:**
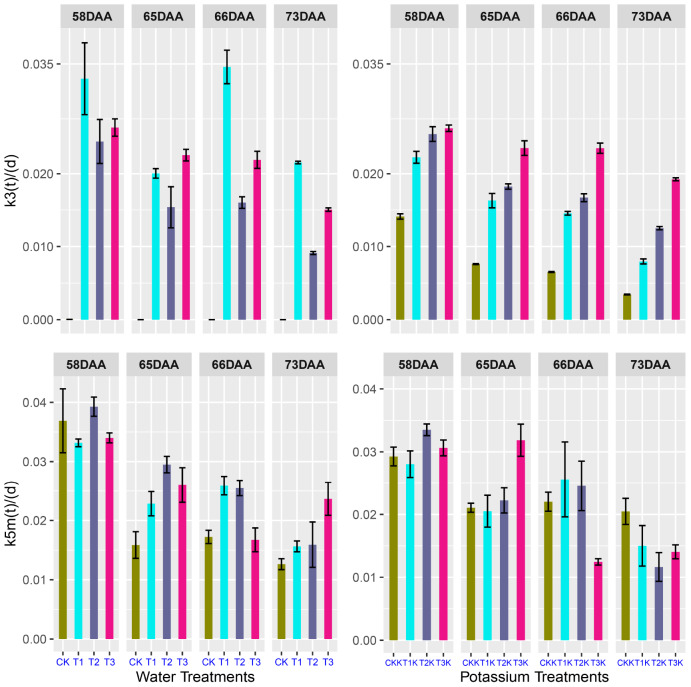
Relative rates of *k*_3_(*t*) and *k*_5*m*_(*t*) at 58, 65, 66, and 73 days after anthesis (DAA) as a function of fruit age. The values were calculated using Eqs 8 and 9 in R project.

Samples were taken at 58, 65, 66, and 73 DAA during fruit maturation. Figure 6 shows changes in the parameters for different treatments. There was little change in *k*_3_(*t*) for CK between 58 and 73 DAA, but for other treatments *k*_3_(*t*) decreased gradually as DAA increased (Figure 6).

*k*_5*m*_(*t*) gradually decreased during fruit maturation for different treatments. The water-deficit treatments showed greater values of *k*_5*m*_(*t*) than CK for 65–73 DAA. CKK showed the least variation (Figure 6), but the change in *k*_5*m*_(*t*) ranged from 0.01 to 0.04 during the fruit ripening stage, which was not considerable. There was very little change in *k*_3_(*t*) and *k*_5*m*_(*t*) during fruit maturation.

## Discussion

Moisture is closely related to plant growth. Tomato plants have a high water demand (Norris, 2006), and tomato fruits are sensitive to water deficit (Rao et al., 2001). The water status of the fruit directly affects the sugar concentration in tomato, so irrigation is an important factor influencing tomato yield and fruit quality. Water stress reduces fruit size in grapes and thus increases the ratio of skin to pulp and improves wine quality (Ginestar et al., 1998; Ojeda et al., 2002; Koundouras et al., 2006). Deficit irrigation of peaches significantly reduces fruit weight but increases soluble solid content (Kobashi, 2000; Génard et al., 2003). Water deficit has a significant effect on fruit weight of melons (Dogan et al., 2008). A study on citrus also confirmed that both the fruit size and single fruit weight decrease in deficit irrigation (Treeby et al., 2007). Clearly, water deficit significantly reduces the weight of fresh fruit. Our experimental data showed that fruit weight for the control CK was greater than for the water deficit treatments (Figure 2 and Table 2), which implies that that water stress in tomatoes decreases FW, which is consistent with the results of the studies mentioned.

Water deficit changes the amount of assimilate received by fruit (Wang et al., 2003), the rate of sugar metabolism (Kobashi, 2000) and the water budget (Keller et al., 2006), thereby altering the sugar content (Mitchell et al., 1991). Water content accounts for about 95% of fruit weight, and dilution is important in determining the concentration of soluble sugars (Génard et al., 2014). The dilution effect (Terry et al., 2007) results in a negative correlation between sugar content and irrigation level (Kobashi, 2000; Sadras and McCarthy, 2008; Julie et al., 2016). In our experiment, total water quantity applied by the treatments was ordered CK > T_1_ > T_3_ > T_2_ (Table 1). T_2_ gave the greatest value of mean SSC and CK the least, demonstrating the dilution effect: as irrigation amount increased, sugar concentration decreased. Thus water deficit increased the concentration of soluble sugars and so improved fruit quality, shown by treatment T_2_ giving the highest SSC.

Fontes et al. (2000) found a correlation between fruit size and potassium content: when potassium was at a low level the fruits were small, and when potassium was at a high level the fruits were large and had thick peels. It was showed that the addition of potassium was significant when the irrigation amount was unchanged. Full irrigation and potassium addition resulted in greatest FW (Table 2). Mean SSC was greater for the K_1_ treatments than for the corresponding K_0_, which shows that SSC increased when potassium was added. We concluded that potassium addition significantly increased SSC. There were no significant differences in STC between treatments (Table 2), possibly due to the lower starch content at fruit maturation. This result was confirmed by the trend of change in the carbon conversion coefficient *k*_5*m*_(*t*), which approached zero at the maturation stage (Figure 4).

Carbon accounts for over 90% of plant dry matter (Zhang et al., 2006). Carbon metabolism is affected by plant genetics and by environmental conditions such as light, temperature, humidity, moisture, and mineral nutrients (Hanson and Roje, 2001; Buckley and Mott, 2013). Water stress influences carbon transport, carbon assimilation, carbon partitioning and carbon metabolism in plants. Our experiment shows that *C*_*sol*_ was significantly less than *C*_*sta*_ during the early development stage (Figure 3) due to the large amount of carbon partitioned for starch synthesis, which resulted in high *C*_*sta*_ content. Colombié et al. (2016) showed that at the beginning of ripening, degradation of starch accumulated during early fruit development is an important source of sugars and energy. It is well known that water stress and salt stress increase starch content in the fruit of some tomato cultivars (Mitchell et al., 1991; Gao et al., 1998; Yin et al., 2010; Biais et al., 2014). We found that water deficit treatment T_1_ resulted in the highest starch content, and that water deficit increased soluble sugar content (Leonardi et al., 2000; Qi et al., 2005). In the early development stage, due to limited sink strength, soluble sugars were stored as starch, which increased starch content, consistent with previous research.

The carbon conversion coefficient *k*_5*m*_(*t*) in our model showed that starch content peaked during the early development stage. The maximum *k*_5*m*_(*t*) value of different water treatments during the early development stage of fruit growth was given by treatment T_1_ (Figure 4). The high initial values of *C*_*sta*_ and *C*_*str*_ gradually decreased and the value of *C*_*sol*_ slowly increased as the fruit developed (Figure 3), indicating that carbon allocation was a dynamic process over the entire fruit growth period and suggesting that there were significant differences in carbon allocation during different growth stages (Table 2). The accumulation of starch and other structural carbon compounds occurred mainly during the early development stage, whereas soluble sugars accumulated mainly during the maturation stage.

The intensity of metabolic changes increases as fruit ripens during the final slow growth period, when glucose and fructose continue to accumulate and the concentration of soluble sugars reaches a maximum. The maturation stage is thus critical to sugar accumulation in the fruit (Prudent et al., 2011). There were few significant differences between indicators over the entire growth stage, but we analyzed the indicators at fruit maturation (Table 2) because understanding the distribution and transformation of carbon during the maturation stage is beneficial to analyzing the final formation process of fruit.

Water had a significant effect on carbon partitioning at fruit maturation. Mean *C*_*sol*_ and *C*_*sta*_ values for water-deficit treatments were significantly greater than for CK, and mean *C*_*str*_ was significantly less (Table 2 and Figure 5). Starch can be hydrolyzed into soluble sugars by the activity of starch phosphorylase and amylase (Smith et al., 2005), which occurs when carbon is converted from *C*_*sta*_ to *C*_*sol*_ in fruit. However, structural carbohydrates cannot be converted into soluble sugars. Thus as *C*_*str*_ increases, *C*_*sol*_ decreases at fruit maturation. In comparison to CK, the water deficit treatments will (1) increase the starch and soluble sugar concentrations during fruit ripening, (2) reduce the content of structural carbon, (3) regulate carbon allocation, and (4) improve fruit quality.

The accumulation of sugar and potassium is closely related to fruit ripening. The mechanism driving this correlation has not yet been elucidated, but it could be that potassium ions increase the efficiency of photosynthesis (Lalonde et al., 2003), which could be coupled to their role in phloem transport (Zelmari et al., 2019). We found that the flux of *C*_*supply*_ (*dC_*sup*_/dt*) was significantly greater when potassium was added over the entire growth stage of the tomato fruit, which is consistent with previous results (Supplementary Figure 2). Figure 5 and Table 2 show that potassium addition reduced *C*_*sta*_ at fruit maturation in treatments T_1_K, T_2_K, and T_3_K, and that *C*_*sol*_ was greater than when potassium was not added. *C*_*str*_ was decreased considerably in potassium treatments, which indicates that potassium addition may regulate carbon allocation and increase the accumulation of soluble sugars, thus increasing *C*_*sol*_ and correspondingly decreasing *C_str_*.

The SUGAR model of sugar metabolism developed by Génard and Souty (1996) was the first to provide a mechanistic representation of biochemical activity during all growth stages (Lobit et al., 2006). Mechanical models are important tools for investigating and understanding carbon allocation within plants (Génard et al., 2008).

Metabolic activity involved in the synthesis of starch and structural carbon compounds decreases during fruit development (Robinson et al., 1988). This observation is confirmed by the decreases in *k*_3_(*t*) and *k*_5*m*_(*t*) as DAA increased (Figure 4); both *k*_3_(*t*) and *k*_5*m*_(*t*) approached zero whether or not potassium was added.

Parameter variation during the critical periods of sugar accumulation can reflect activity during the fruit growth stage. For instance, sugar transported from the source to the sink during the early growth stage is mainly used for synthesis of structural compounds such as cellulose and protein (Keller and Steffen, 1995), as well as other structural compounds in the cell, to maintain normal cell metabolism. The maximum value of *k*_3_(*t*) was found during the early fruit development stage. Sugar and starch were gradually accumulated during the fruit development stage; however, sink strength was limited, and the concentration of structural carbon decreased. Thus, *k*_3_(*t*) was bound to decrease (Figure 4).

Starch accumulation reached a maximum and *k*_5*m*_(*t*) also reached a maximum during the interval 25–30 DAA. The coefficient of correlation between starch concentration and *k*_5*m*_(*t*) reached 0.93 (Sweetlove et al., 1999). *k*_3_(*t*) and *k*_5*m*_(*t*) decreased as DAA increased over the entire growth period and reached minimum values at fruit maturation.

## Conclusion

With the assistance of the SUGAR model, we analyzed the effects of water and potassium supply on carbon allocation and conversion of different carbohydrates as indicated in tomato fruit.

The results showed that carbon allocation and transformation changed dynamically during all growth stages, but that the trend of the variation was the same for different water and potassium treatments. The results also showed that the growth stage had a significant effect on carbon allocation. Starch accumulation and the formation of structural carbon compounds were the main forms of carbon found in the early fruit growth stage, and *k*_3_ and *k*_5*m*_ peaked during this stage. The starch was eventually converted to soluble sugars, and soluble sugars were the main form of carbon found in the fruit at fruit maturation, when *k*_3_(*t*) and *k*_5*m*_(*t*) decreased to zero.

Potassium addition significantly increased the formation of soluble sugars over the entire growth stage. Potassium addition decreased both *C*_*sta*_ and *C*_*str*_ during fruit maturation compared to no potassium addition.

We concluded that water deficit regulated carbon allocation in tomato fruit and increased soluble sugar content. Potassium application noticeably increased carbon flow from phloem into the fruit. The interactive effect of water and potassium on *C*_*str*_ during fruit maturation was significant. Both water deficit and potassium application decreases the content of structural carbon in the fruit, thereby improving fruit quality.

## Data Availability Statement

All datasets generated for this study are included in the article/Supplementary Material.

## Author Contributions

AL did the experiment and finished the first manuscript. SK supervised the work. JC helped to do the experiment.

## Conflict of Interest

The authors declare that the research was conducted in the absence of any commercial or financial relationships that could be construed as a potential conflict of interest.

## Supplementary Material

FIGURE S1 Details of the experiment site and plant layout in the greenhouse.

FIGURE S2 Diurnal variation of carbon supply flux between potassium and without potassium treatments during the all growth stages.
