# Supplementary figures and images for: SUGAR Model-Assisted Analysis of Carbon Allocation and Transformation in Tomato Fruit Under Different Water Along With Potassium Conditions
Source: Front Plant Sci. 2020 Jun 5;11:712. doi: 10.3389/fpls.2020.00712 (PMC7292204; doi:10.3389/fpls.2020.00712)

SUPPLEMENTARY FIGURE 1

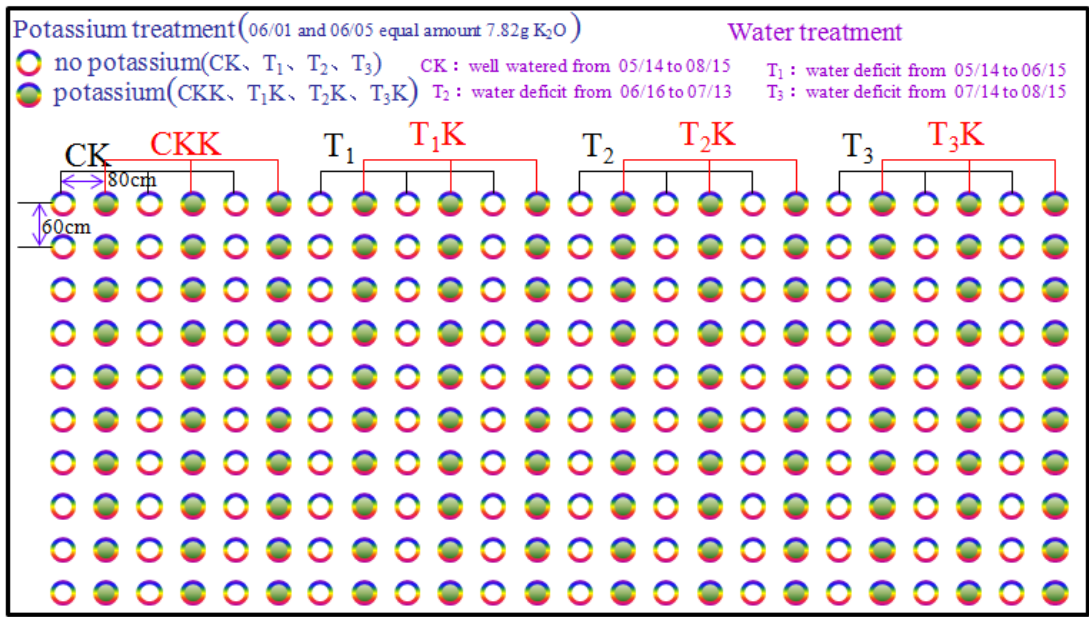

Supplement: FIGURE S1 — Details of the experiment site and plant layout in the greenhouse. [file Image_1.pdf]

## SUPPLEMENTARY FIGURE 2

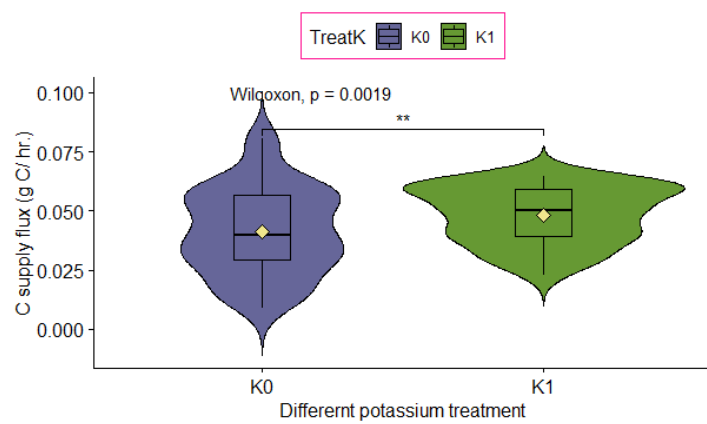

Supplement: FIGURE S2 — Diurnal variation of carbon supply flux between potassium and without potassium treatments during the all growth stages. [file Image_2.pdf]
